# EMPOWERING FALLS PREVENTION IN OLDER ADULTS: INTENTIONAL ACADEMIC–COMMUNITY PARTNERSHIPS FOR SUCCESS

**DOI:** 10.1093/geroni/igae098.0296

**Published:** 2024-12-31

**Authors:** Sara Pappa

**Affiliations:** Marymount University, Arlington, Virginia, United States

## Abstract

The Northern Virginia Falls Prevention Alliance exemplifies a robust academic-community partnership for deploying evidence-based falls prevention programs (EBFPP) within a metropolitan setting. The project provides accessible EBFPP such as “Stay Active and Independent for Life” (SAIL) for those at low to moderate risk, “A Matter of Balance” (MOB) aimed at alleviating the fear of falling, and the “Otago Exercise Program” (OEP) for high-risk individuals. Over eight years, the program has engaged more than 6,000 older adults, spread across many Northern Virginia community-based locations, showcasing a significant breadth of reach and adaptability. Adding to this, the recent incorporation of the “Saving Claire” Falls Prevention Project documentary heightens awareness and education, bolstering the program’s outreach. This presentation will emphasize the pivotal roles of collaborative community organization, sustainable program delivery, leader preparation, and fostering trust with older adults, thereby reinforcing public health endeavors to mitigate fall-related injuries. With over a decade of program evolution, this session will outline key strategies for sustainability, accessibility, and adoption. Emphasizing the practicality and impact of academic-community alliances, it will underscore how such partnerships are instrumental in advocating the successful uptake and sustainability of EBFPP

